# *In Vitro* and *in Vivo* Inhibitory Effects of Glycyrrhetinic Acid in Mice and Human Cytochrome P450 3A4

**DOI:** 10.3390/ijerph13010084

**Published:** 2015-12-25

**Authors:** Qiao-Li Lv, Gui-Hua Wang, Shu-Hui Chen, Lei Hu, Xue Zhang, Guo Ying, Chong-Zhen Qin, Hong-Hao Zhou

**Affiliations:** 1 Department of Clinical Pharmacology, Xiangya Hospital; Institute of Clinical Pharmacology, Hunan Key Laboratory of Pharmacogenetics, Central South University, Changsha 410008, China; lvqiaoli2008@126.com (Q.-L.L.); zhuifenglang@163.com (L.H.); zhangx2013cs@163.com (X.Z.); guoying198688@126.com (G.Y.); 2 Department of Oncology, Changsha Central Hospital, Changsha 410006, China; wangguihua2008@126.com (G.-H.W.); chenshuhui2008@126.com (S.-H.C.); 3 Department of Pharmacy, The First Affiliated Hospital of Zhengzhou University, Zhengzhou 450052, China

**Keywords:** glycyrrhetinic acid, cytochrome P450 3A4, inhibitory effect, herb–drug interaction, IC_50_

## Abstract

Glycyrrhetinic acid (GA) has been used clinically in the treatment of patients with chronic hepatitis. This study evaluated the effect of GA on the activity of five P450(CYP450) cytochrome enzymes: CYP2A6, CYP2C9, CYP2C19, CYP2D6, and CYP3A4, in human liver microsomes (HLMs) and recombinant cDNA-expressed enzyme systems using a HPLC-MS/MS CYP-specific probe substrate assay. With midazolam as the probe substrate, GA greatly decreased CYP3A4 activity with IC_50_ values of 8.195 μM in HLMs and 7.498 μM in the recombinant cDNA-expressed CYP3A4 enzyme system, respectively. It significantly decreased CYP3A4 activity in a dose- but not time-dependent manner. Results from Lineweaver–Burk plots showed that GA could inhibit CYP3A4 activity competitively, with a Ki value of 1.57 μM in HLMs. Moreover, CYP2C9 and CYP2C19 could also be inhibited significantly by GA with IC_50_ of 42.89 and 40.26 μM in HLMs, respectively. Other CYP450 isoforms were not markedly affected by GA. The inhibition was also confirmed by an *in vivo* study of mice. In addition, it was observed that mRNA expressions of the *Cyps2c* and *3a* family decreased significantly in the livers of mice treated with GA. In conclusion, this study indicates that GA may exert herb-drug interactions by competitively inhibiting CYP3A4.

## 1. Introduction

Natural compounds are used in the treatment of refractory diseases more and more frequently because of their efficacy and low toxicity. The traditional Chinese medicine *Gancao* (licorice root) is a crude drug of *Glycyrrhiza uralensis* Fisch (licorice), G. glabra L. var. glandulifera Waldst. and Kit, is believed to harmonize formulations to carry the formula to the 12 regular meridians [1]. Numerous studies have revealed many pharmacological activities of licorice, such as antimicrobial [2], antiviral [3,4] antitumor [5,6], anti-inflammatory [7,8], and many other activities [9,10]. It is reported that licorice contains nearly 300 flavonoids and more than 20 triterpenoids, among which glycyrrhetinic acid (GA), a pentacyclic triterpene acid with numerous biological activities, has anti-inflammatory [11,12], antiviral [13], antiallergic [14], and antitumor proliferative effects [15,16].

Recently, several *in vitro* studies in rat or human liver microsomes have demonstrated that CYP3A and CYP2C9 enzymes participate in the 22α- and 24-hydroxylation of GA, respectively [17,18]. Moreover, the activities of CYP2C9, CYP2C19 and CYP3A4 enzymes were inhibited by GA by 50% [17,18]. Induction and inhibition of cytochrome P450 (CYP) enzymes and drug transporters by traditional chinese medicine (TCM) result in changes of their substrate pharmacokinetic parameters, mediating herb-drug interactions, and producing clinical drug toxicity. Therefore, to clarify the underlying mechanism of herb-drug interactions has great clinical significance, which can provide important information on the source that contributes to adverse drug reactions (ADRS), guide rational drug use and avoid the generation of ADRs occurring from a herbal-drug interaction. Currently, the inhibition mode of GA is not clear and as there are no *in vivo* studies validating the herbal-drug interaction.

In the present study, we evaluated the mechanism of inhibition mode of GA through cytochrome P450 isoforms (CYP450) and which confirmed an inhibitory effect in mice. The study provides evidence that a herbal-drug interaction may occur, depending on the drug or class of drugs administered, resulting from CYP450 inhibition.

## 2. Materials and Methods

### 2.1. Chemicals

The pooled human liver microsomes (HLMs) and human recombinant enzyme used in the incubation studies were purchased from BD Gentest Co. (Woburn, MA, USA). Coumarin, 7-hydroxycoumarin, tolbutamide, 4-hydroxytolbutamide, (S)-mephenytoin, 4′-hydroxymephenytoin, metoprolol, α-hydroxymetoprolol, midazolam maleate, 1-hydroxymidazolam, propranolol, quinidine hydrochloride, tranylcypromine, fluconazole, ticlopidine, quinidine, ketoconazole, furafylline, and diethyldithiocarbamate were purchased from Sigma Chemicals (St. Louis, MO, USA). Formic acid, ethylic acid, ammonium formate, MgCl_2_, NADP^+^, glucose 6-phosphate, glucose 6-phosphate dehydrogenase, and potassium phosphate (monobic and dibaic) were chromatographic-grade chemicals purchased from Sigma Chemicals (St. Louis, MO, USA). Acetonitrile and methanol were of analytical grade and purchased from Sigma Chemicals (St. Louis, MO, USA). PrimeScript^®^ RT reagent Kit With gDNA Eraser and SYBR^®^ Premix DimerEraser™ were purchased from Takara Biotechnology (Dalian, China). Glycyrrhetinic acid (GA) was purchased from National Institute for the Control of Pharmaceutical and Biological Products (Beijing, China).

### 2.2. Animals and Treatments

Eight-week old male C57BL/6 mice were obtained and maintained at the Animal Experimental Center of Zhengzhou University according to the Institutional Animal Care Guidelines in a light-dark (12 h–12 h), temperature (23 °C ± 2 °C) and humidity (50% ± 8%) controlled laboratory environment. Sterilizing water and standard diet were provided for the animals, and standard iron cages were used for six mice per cage. GA (0, 25, 50, and 100 mg/kg, once daily) was administered to mice (*n* = 5 for each dose) for 15 days. After the end of study, mice were killed rapidly, decapitated to collect livers frozen in liquid nitrogen, and stored in −80 °C for further assays. The study was approved by the ethical committee of the Zhengzhou University (No. SYXK-2012-0001) and complied with national laws relating to the conduct of animal experiments.

### 2.3. Total RNA Extraction and Real-Time PCR Quantification

Total RNA was extracted from frozen livers using TRIzol reagent. The purity and concentration of the RNA were calculated by measuring its absorbance at 260 and 280 nm. A PrimeScript^®^ RT reagent Kit was used to synthesize cDNA from 1 μg RNA. Primers for related genes were chosen and are in Table 1. Real-time RT-PCR was conducted using SYBR^®^ Premix DimerEraser™ on a LightCycler^®^ 480 (Roche Diagnostics, Basel, Switzerland) detection system. The mRNA gene expression levels were normalized to β-actin gene expression.

**Table 1:** Primer sequences for real-time PCR reactions.

Target	Accession Number	Forward Primer	Reverse Primer
Cyp2a4	NM_009997	GGAAGACGAACGGTGCTTTC	CCCGAAGACGATTGAGCTAATG
Cyp3a11	NM_007818	CGCCTCTCCTTGCTGTCACA	CTTTGCCTTCTGCCTCAAGT
Cyp2c37	NM_010001	ATACTCTATATTTGGGCAGG	GTTCCTCCACAAGGCAAC
Cyp2c39	NM_010003	GGAGACAGAGCTGTGGC	TAAAAACAATGCCAAGGCCG
Cyp2d22	NM_019823	GGGCCTTTGTTACCATGTTGG	TACTCGGCGCTGCACATCTG
β-actin	NM_007393	TGTTACCAACTGGGACGACA	GGGGTGTTGAAGGTCTCAAA

### 2.4. Liver Microsome Preparation and Determination of Cyp Activity

Microsomes were prepared from each mouse liver according to the method of Zhao and colleagues [19]. Protein concentrations were determined following the manufacturer's instructions using a Pierce^®^ BCA Protein Assay Kit (Thermo Scientific, Rockford, IL, USA).

A previous validated method was followed for incubation of liver microsome, NADPH regeneration, sample preparation, and HPLC-MS/MS analysis [20].

### 2.5. Inhibitory Effects of GA on Cytochrome P450s

Compound assays were performed in triplicate using individual drug-probe substrates specific for each HLM-CYP or recombinant enzyme with HPLC-MS/MS detection. The concentration range of the inhibitors was set from 0.01 μM to 100 μM (five points).

### 2.6. Enzymatic Kinetic Study of Inhibition

To investigate the inhibitory mode of GA, HLMs were pre-incubated with GA in potassium phosphate buffer (pH 7.4) for 0, 10, or 30 min in the presence of NADPH. Midazolam was then added and incubated for 30 min at 37 °C. Midazolam was used as a probe substrate at 5, 10, or 20 μM.

### 2.7. cDNA-Expressed CYP3A4 Inactivation Assay

To confirm the selective inhibition of CYP3A4 enzyme by GA, 10 pmol of human recombinant cDNA-expressed CYP3A4 was incubated with GA at 0.01 to 100 μM, with the NADPH system and midazolam as a selective CYP3A4 substrate for 30 min at 37 °C.

### 2.8. Statistical Analysis

A value of *p* < 0.05 was considered statistically significant. The 50% inhibitory concentration (IC_50_) values were calculated by nonlinear regression using GraphPad Prism 5.0 (San Diego, CA, USA). Lineweaver–Burk plots were obtained from SigmaPlot software (version 10.0, Systat Software, Inc.: Chicago, IL, USA).

## 3. Results

### 3.1. Inhibition of CYP450 Activity by GA in HLMs

**Figure 1:**
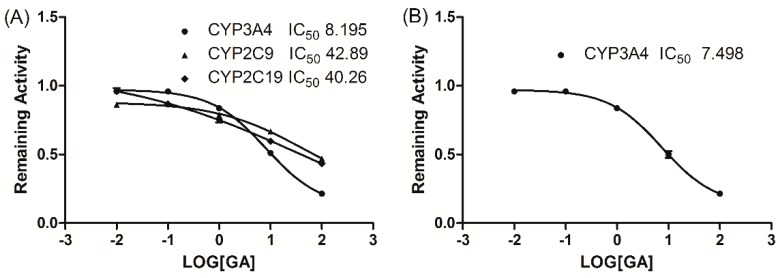
The inhibitory effect of GA (0, 0.01, 0.1, 1, 10, and 100 μM) on CYP3A4, CYP2C9 and CYP2C19 enzyme activity in HLMs (**A**) and on the midazolam 1′-hydroxylation activities of human recombinant cDNA-expressed CYP3A4 (**B**).The data were expressed as the mean ± SD.

The inhibitory effects of GA on five typical reactions catalysed by five human CYP enzymes were examined in HLMs. GA showed potent inhibition of CYP3A4 activity in HLM with an IC_50_ value of 8.195 μM, and a moderate inhibition of CYP2C9 and CYP2C19 activity was observed with IC_50_ values of 42.89 and 40.26 μM, respectively. However, GA caused no significant inhibition of CYP2A6, CYP2D6, or CYP2E1 activity (IC_50_ > 100 μM). Figure 1A illustrates the inhibitory effects of GA on CYP3A4, CYP2C9, and CYP2C19 in HLMs.

### 3.2. Mechanism-Based Inhibition of CYP3A4 by GA

**Figure 2:**
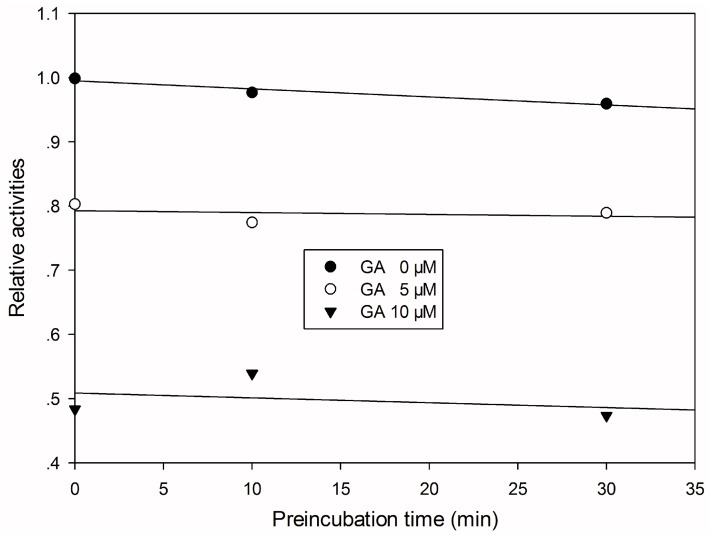
Time-course of CYP3A4-catalyzed midazolam 1′-hydroxylation inactivation by GA in HLMs. HLMs were preincubated with GA at 0 (●), 5 (○), or 10 (▼) μM in the presence of NADPH for 0 to 30 min.

**Figure 3:**
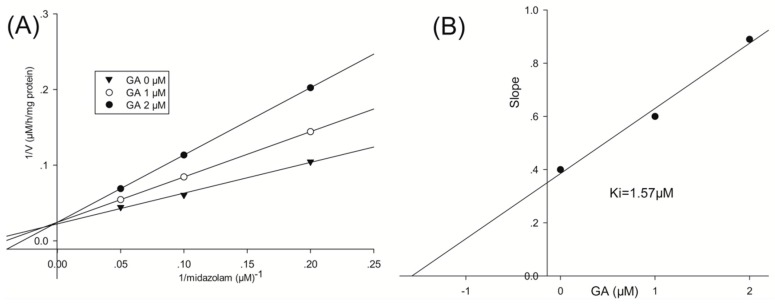
Lineweaver–Burk plot (**A**) and secondary plot (**B**) obtained from a kinetic study of CYP3A4-catalyzed midazolam 1′-hydroxylation following a 30 min incubation with GA at 0 (▼), 1 (○), or 2 (●) μM.

To investigate the mode by which GA inhibits CYP3A4-catalyzed midazolam 1′-hydroxylation, CYP3A4 inhibitory activity was determined with or without pre-incubating microsomal mixtures for 0, 10, or 30 min at 37 °C in HLMs. GA strongly and dose-dependently inhibited CYP3A4-catalyzed midazolam 1′-hydroxylation in HLMs, but not time-dependently (Figure 2). To investigate the mechanism of inhibition of CYP3A4 by GA (0, 1, or 2 μM), different concentrations (5, 10, or 20 µM) of midazolam were tested. From Lineweaver–Burk plots (Figure 3A) and secondary plots (Figure 3B), both of which were linear, a competitive type of inhibition by GA on CYP3A4 was observed. Based on the observations derived from these experiments, we conclude that GA acts as a competitive inhibitor of CYP3A4 with a Ki value of 1.57 μM in HLMs.

### 3.3. Inhibition of Recombinant CYP3A4 by GA

To evaluate the selectivity of the inhibitory effect of GA on CYP3A4, GA was incubated with human recombinant cDNA-expressed CYP3A4. GA showed potent inhibition of CYP3A4 activity with an IC_50_ value of 7.498 μM (Figure 1B).

### 3.4. Inhibition of CYPs by GA in Mice

**Figure 4:**
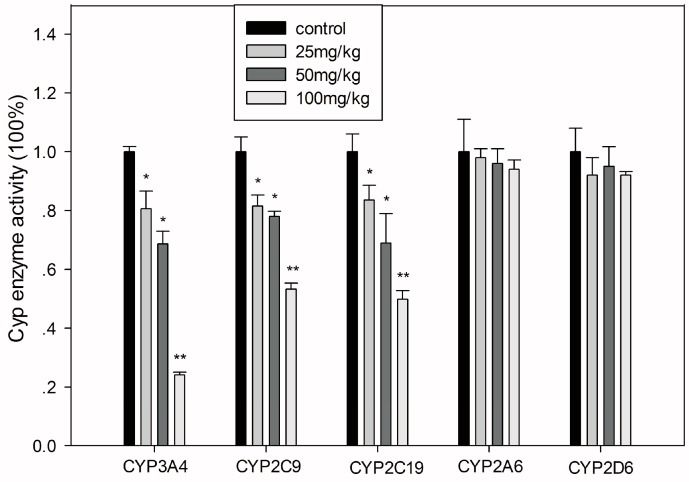
Inhibition of major hepatic drug metabolizing enzymes in mice treated with various doses of GA. All values are presented as the mean ± SD; * *p* < 0.05; ** *p* < 0.01.

After treatment with different concentrations of GA for 15 days, the activity of different CYPs in mouse liver were measured through metabolites of CYP isotype-specific probe drugs. Consistent with the results of the *in vitro* study in HLMs, CYP3A4, CYP2C9, and CYP2C19 were each inhibited significantly in a GA dose-dependent manner (Figure 4).

### 3.5. Effects of GA on the mRNA Expression Levels of CYPs

**Figure 5:**
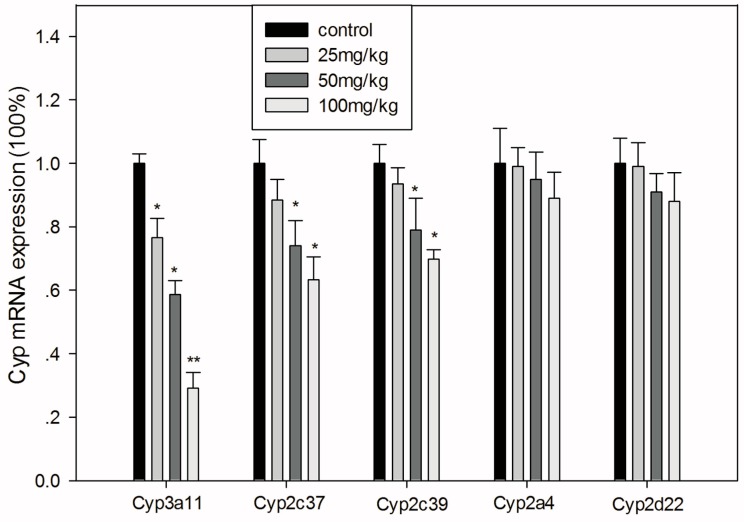
Relative mRNA expression levels of major hepatic Cyps in mice treated with GA. All values are presented as the mean ± SD; * *p* < 0.05; ** *p* < 0.01.

The mRNA expression levels of genes encoding Cyps are shown in Figure 5. Cyp3a11 decreased dose-dependently and more markedly than the other Cyps after GA treatment. Cyp2c37 and Cyp2c39 decreased significantly when mice were treated with 50 or 100 mg/kg GA (*p* < 0.05), while the lower dose of GA only showed a slight decreasing tendency (*p* > 0.05). Moreover, GA showed no statistically significant effect on the expression of Cyp2a4 or Cyp2d22 (*p* > 0.05).

## 4. Discussion

This study is the first to investigate the enzyme kinetics of the inhibitory effects of GA on CYP450 enzymes activities in HLMs, as well as in mice. GA significantly inhibits CYP3A4, CYP2C9, and CYP2C19 enzyme activity in HLMs and in mice. Moreover, GA significantly decreased the mRNA expression of Cyp 2c and 3a family members in mice.

CYP3A4 is responsible for the metabolism of a variety of drugs and endogenous compounds in humans. CYP3A4 constitutes up to 60% of liver CYP isoforms. Over 50% of marketed drugs are metabolized by CYP3A4 [21,22]. At present, chronic hepatitis could be treated by numerous drugs, such as IFN or pegylated IFN, and numerous nucleoside analogues including lamivudine, telbivudine, clevudine, entecavir, adefovir dipivoxil and tenofovir dipivoxil fumarate [23,24,25]. The metabolism of many of these drugs involves the activity of CYP3A4, CYP2C9, or CYP2C19.

GA, one of the bioactive compounds from *Gancao*, has been widely used in the treatment of inflammatory diseases, especially liver disease. Complications may occur when patients are treated with drugs concomitantly with GA. Serious or even fatal adverse drug reactions (ADRs) may occur owing to alteration of CYP activity by drugs concomitantly with GA [26]. Herbal-drug interaction between GA and other drugs especially those known to be metabolized by CYP3A4 should be taken with caution.

There have been several studies about the metabolism of GA [17,18], indicating that CYP3A and CYP2C9 enzymes play a vital role in the 22α- and 24-hydroxylation of GA, respectively. Another study from Zhao *et al.* [18] indicated that CYP3A4 is the major enzyme responsible for GA metabolism, whereas CYP2C9 and CYP2C19 are minor ones. Interestingly, they also observed that GA inhibited the 1-hydroxylation of midazolam by CYP3A4, the 4-hydroxylation of diclofenac by CYP2C9 and the 4′-hydroxylation of (S)-mephenytoin by CYP2C19. The results were based on rat and human microsomes, but only *in vitro* evidence was provided. A study of Liu *et al.* [17] evaluated oxidation activity toward GA in HLMs, the metabolism of GA and the effects of GA on the activity of main CYP isoforms. These studies have enhanced our knowledge of the pharmacokinetics of GA and their effects on drug metabolism. However, which type of competitive inhibition was not elucidated. Our study supplemented the knowledge of GA inhibition kinetics and provided *in vivo* evidence of its inhibition of CYPs. In this study, GA showed potent competitive inhibition of CYP3A4 activity. No significant inhibition of CYP2A6, CYP2D6, or CYP2E1 activity was noted.

GA could induce the activation of PAI 1 expression and NaDPH oxidase through direct binding to mineralocorticoid receptors [27]. The CYP3A locus, including all the known members of the 3A subfamily of the cytochrome P450 superfamily of genes, encode monooxygenases which catalyze many reactions involved in drug metabolism and synthesis of cholesterol, steroids, and other lipids [28]. Steroids can have a specific inflammatory or anti-inflammatory effect, so our study could imply that GA may change the inflammatory or anti-inflammatory effect by inhibiting CYP3A activity.

The results provide *in vitro* and *in vivo* evidence of potential drug interactions involving GA. Further clinical trials are needed to determine whether GA exerts adverse effects when co-administered with other drugs. Whether it exerts a favourable influence on other medical treatments also deserves further investigation.

## 5. Conclusions

This study demonstrated an inhibitory effect of GA on CYP3A4-catalyzed midazolam 1′-hydroxylation in that acts HLMs in a competitive manner. Moreover, GA may also inhibit CYP2C9 and CYP2C19 activity. This inhibition was also validated by an *in vivo* study in which the mRNA expression of Cyp 2c and 3a family members in mice was decreased significantly by GA. The result indicated that GA might cause herbal–drug interactions when co-administrated with substrates of CYP3A4, CYP2C9, and CYP2C19.
